# Benzodiazepine use among adults residing in the urban settlements of Karachi, Pakistan: A cross sectional study

**DOI:** 10.1186/1747-597X-6-19

**Published:** 2011-08-01

**Authors:** Saleem P Iqbal, Syed Ahmer, Salima Farooq, Yasmin Parpio, Ambreen Tharani, Rashid AM Khan, Mohammad Zaman

**Affiliations:** 1 Department of Pediatrics & Child Health, Aga Khan University, Stadium Road, Karachi, Pakistan; 2 Department of Psychiatry, Aga Khan University, Stadium Road, Karachi, Pakistan; 3 School of Nursing, Aga Khan University, Stadium Road, Karachi, Pakistan

## Abstract

**Background:**

There are hardly any studies carried out in Pakistan on the usage of benzodiazepines at the level of community. This research was aimed to determine the frequency of benzodiazepine use, along with its associations with socio-demographic and clinical characteristics among community dwelling adults, residing in two urban settlements of Karachi, Pakistan.

**Methods:**

We performed a cross sectional study from August 2008 to December 2009, in 2 areas of Karachi, namely Garden and Sultanabad. We followed the systematic sampling strategy to randomly select the households, with an adult of either sex and of age 18 years or more. Data collection was carried out through interview, using a pre-tested questionnaire, with items on socio-demographic position, medical history and benzodiazepine use. Student's t-test and χ^2 ^test was employed to determine the associations between socio-demographic and clinical characteristics, and their relationship with benzodiazepine use was determined using applied logistic regression.

**Results:**

The overall percentage of benzodiazepine consumption was estimated to be 14%. There were significantly more benzodiazepine users in the peri-urban Sultanabad community to the urban community of Garden (p-value = 0.001). The mean age (± SD) for users was 51.3 (± 15.6) years compared to 37.1 (± 14.4) years among non-users. Bromazepam was the most widely used benzodiazepine (29%); followed by diazepam, with a median duration on primary use being 144 weeks (IQR = 48-240). The adjusted logistic regression model revealed that increasing age, location, female sex, unemployment and psychiatric consultation were associated with increased likelihood of benzodiazepine use.

**Conclusion:**

We believe the unregulated over-the-counter sales of benzodiazepines and social conditions might be playing a role in this high consumption of benzodiazepines in the community.

## Background

After the discovery of chlordiazepoxide in 1957, benzodiazepines are among the most widely used drugs in the general population [1-6]. These were generally prescribed to combat anxiety and insomnia but over time these are now being used for a wide array of psychiatric and non-psychiatric illnesses, in view of their sedative, anti-convulsant and muscle relaxing properties [1,7-9].

Despite their therapeutic uses, tendency remains for benzodiazepine abuse among the general population. Several adverse reactions have been reported in studies which include cognitive decline, motor disturbances, unwanted sedation and reduced coordination [10,11]. In addition, benzodiazepines pose a risk of dependence which may become as high as 45% after 6 months of continuous use [12,13]. In view of this, benzodiazepine sale and usage have been regulated in many developed countries [14]. However, this is not commonly practiced in most developing countries, including Pakistan, where practically these can be purchased over-the-counter, with considerable ease [9,15,16].

Keeping in view the various definitions of benzodiazepine use, types of population and settings, and periods of observation, estimates reveal that prevalence of benzodiazepine consumption may range from 2% to 17% [17,18]. A recent summary from USA reports a 12-month prevalence of use of benzodiazepines (as tranquilizers) as 8.6% [19]. In a local study, the percent use of benzodiazepines among in-patients in a tertiary care hospital in Pakistan is reported to be 21.2% [16]. In another recent study on outpatients in 2 tertiary care hospitals, use of benzodiazepines is reported to be around 45% [20].

There are hardly any studies in this region to estimate the frequency of benzodiazepine use at the level of community. Most studies in the Western countries have been conducted on elderly populations (more than 65 years) where the prevalence estimates have been found to be 9.9% in USA, 20% in Canada and 31.9% in France [21-23]. From a study in Brazil, a 22% frequency of benzodiazepine use has been reported among elderly persons living in the community [24,25]. Few studies have been carried out in the general population even in the developed countries. For example studies from Boston, France and Lebanon benzodiazepine usage has been reported to be 7.3%, 7.5% and 9.9%, respectively [26-28]. In another study a prevalence estimate of 9.2% benzodiazepine usage was reported from a combined population of 6 European countries [29]. Such estimates are not available for a developing country like Pakistan, where we might expect these to be much higher owing to easy accessibility of these drugs, without a prescription.

Risk factors, common to benzodiazepine use are increasing age and female gender, and have been reported in most studies, regardless of the population [16,20,22,24,25,27,28,30]. Other reported associations are cigarette smoking, marital status, education and occupation [16,27,28]. The objective of the present study was to estimate the frequency of benzodiazepine use among 2 populations in Karachi, Pakistan, and to determine its associations with related socio-demographic and clinical characteristics which are unique to these populations.

## Methods

### Study Design and settings

A cross sectional study was conducted from August 2008 to December 2009. Two sites from the metropolitan city of Karachi, Pakistan were selected for this research, namely Sultanabad and Garden, as community health services were provided to these areas by the Aga Khan University. Moreover, there were differences in the socio-economic conditions in these 2 areas.

Sultanabad is a relatively low income squatter settlement situated in the district East of Karachi. Mainly inhabited by the Pashtoon and Punjabi migrants from northern Pakistan, it has a population of more than 53000 people. Garden is one of the neighborhoods adjacent to the Central district of Karachi. Estimated population is more than 65000 and majority of the people belong to a relatively affluent group. Due to these reasons, these places were well suited to have a comparative assessment of benzodiazepine use among the community people.

### Sampling strategy

We used a systematic random sampling approach to randomly select a representative sample from each of the study sites. About 4000 households from Sultanabad and about 5000 households from Garden were available for this study. Employing the strategy, every 10^th ^household was approached for the interview starting from a random pick using random number tables, from the first 10 households. Our inclusion criteria were to select an adult of age 18 years and above of either sex. If the subject from a household was found unwilling to participate in the study, the adjacent house was approached.

### Data Collection

Data were collected using a specially designed questionnaire, similar to the questionnaire used in a previously published study (20). The participation by the subjects was voluntary. At first they were inquired about their demographic and socioeconomic position such as age, gender, marital status, ethnicity, education and occupation. Following that, questions related to any medical and psychiatric ailments, any consultations and if any medications used were asked. If benzodiazepines were found in use, the subjects were asked whether it was self medication or prescribed. In addition, the duration, frequency, dose and route of administration were noted. Equivalent doses were later calculated using the Maudsley Prescribing Guidelines [31].

### Statistical Analysis

Analyses were performed using the Statistical Package for Social Sciences for Windows version 17 (SPSS Inc., Chicago, IL., USA). Frequencies were calculated for all characteristics and expressed in either means (± SD) or medians (IQR) for quantitative variables and percentages for categorical characteristics. Chi-square (χ^2^) of independence was used to determine significant associations between categorical variables, whereas t-test was employed to assess the differences among quantitative characteristics. Applied logistic regression was used to determine the factors predicting the likelihood of benzodiazepine use. P-value, if less than 0.05 was considered significant.

### Ethical approval

Informed consent was obtained before interviewing the subjects for the study. They were told that their participation was voluntary and they were at liberty to refuse if they wished so. The study was approved by the Ethics Review Committee (ERC) of the Aga Khan University.

## Results

Tables 1 and 2 summarize the socio-demographic and clinical characteristics with respect to benzodiazepine usage in the overall sample and at the 2 sites, respectively. A total of 749 subjects were recruited, out of which 102 (14%) were currently using benzodiazepines. Benzodiazepine users was older compared to the non-users (mean age 51.3 ± 15.6 years vs. 37.1 ± 14.4 years; t = -9.1; df = 746; p-value < 0.001). Significantly more females compared to males were using benzodiazepines (17% vs. 9%; Wald χ^2 ^= 8.1; df = 1; p-value = 0.004).

**Table 1:** Characteristics of the study population with respect to benzodiazepine use (n = 749)

Characteristics		Total	Benzodiazepine users	Wald χ^2^	df	P-value	OR (95% CI)
**Age (means ± SD)**		38.96 ± 15.33	51.30 ± 15.58	62.7	1	0.001	1.06 (1.04-1.07)

**Location**	Garden, n (%)	383	33 (9)				1
	Sultanabad, n (%)	366	69 (19)	15.9	1	0.001	2.46 (1.58-3.84)

**Gender**	Male, n (%)	349	33 (9)				1
	Female, n (%)	399	67 (17)	8.4	1	0.004	1.93 (1.24-3.01)

**Marital Status**	Unmarried, n (%)	249	12 (5)				1
	Married, n (%)	500	88 (18)	20.5	1	0.001	4.22 (2.26-7.87)

**Education**	Post-graduation, n (%)	71	5 (7)				1
	Graduation, n (%)	213	17 (8)	19.5	1	0.001	1.15 (0.41-3.22)
	Intermediate, n (%)	171	13 (8)	6.3	1	0.01	1.09 (0.37-3.17)
	Secondary, n (%)	176	21 (12)	1.3	1	0.26	1.79 (0.65-4.94)
	Primary, n (%)	35	9 (26)	0.02	1	0.9	4.57 (1.40-14.93)
	None, n (%)	72	31 (43)	0.07	1	0.8	9.98 (3.59-27.73)

**Occupation**	Student, n (%)	98	4 (4)				1
	Salaried workers, n (%)	128	17 (13)	5	1	0.03	3.60 (1.17-11.07)
	Housewife, n (%)	195	44 (23)	12.8	1	0.001	6.85 (2.38-19.68)
	Retired, n (%)	31	9 (29)	12.3	1	0.001	9.61 (2.71-34.10)
	Labor, n (%)	197	14 (7)	1	1	0.313	1.80 (0.58-5.61)
	Business, n (%)	81	6 (7)	0.9	1	0.342	1.88 (0.52-6.91)
	Unemployed, n (%)	10	5 (50)	15.1	1	0.001	23.50 (4.78-115.59)

**Psychiatric consult**	No, n (%)	703	75 (11)				1
	Yes, n (%)	36	22 (61)	50.4	1	0.001	13.16 (6.46-26.81)

SD = Standard deviation; df = degrees of freedom; OR = Odds ratio; CI = Confidence interval

**Table 2:** Characteristics of the study population with respect to location

Characteristics		Sultanabad (n = 366)	Garden (n = 383)	Test	df	P-value
**Age (means ± SD)**		38.09 ± 15.3	39.81 ± 15.3	t = -1.53	743	0.13

**Gender**				χ^2 ^= 3.2	1	0.08
	Male, n (%)	157 (43)	190 (50)			
	Female, n (%)	206 (57)	192 (50)			

**Marital Status**				χ^2 ^= 4.4	1	0.04
	Unmarried, n (%)	134 (38)	113 (30)			
	Married, n (%)	230 (62)	269 (70)			

**Education**				χ^2 ^= 31.9	5	0.01
	Post-graduation, n (%)	32 (9)	38 (10)			
	Graduation, n (%)	114 (32)	98 (26)			
	Intermediate, n (%)	67 (19)	104 (27)			
	Secondary, n (%)	70 (20)	106 (28)			
	Primary, n (%)	28 (8)	7 (2)			
	None, n (%)	43 (12)	28 (7)			

**Occupation**				χ^2 ^= 34.5	6	0.01
	Student, n (%)	50 (14)	47 (12)			
	Salaried workers, n (%)	90 (25)	38 (10)			
	Housewife, n (%)	78 (22)	117 (31)			
	Retired, n (%)	12 (3)	19 (5)			
	Labor, n (%)	93 (26)	104 (27)			
	Business, n (%)	33 (9)	48 (13)			
	Unemployed, n (%)	3 (1)	6 (2)			

**Psychiatric consult**				χ^2 ^= 0.82	1	0.36
	No, n (%)	346 (96)	354 (94)			
	Yes, n (%)	15 (4)	21 (6)			

SD = standard deviation

df = degrees of freedom

The mean ages between male and female benzodiazepine users were not significantly different (mean age 51.6 ± 14.2 years vs. 50.8 ± 16.2 years; t = 0.24; df = 93; p-value = 0.81). Majority of the people in the sample were married (67%), and 18% of them were consuming benzodiazepines. Benzodiazepine use was highest (43%) in the category of uneducated persons. In terms of frequency of benzodiazepine use in the 2 communities, there is significantly greater proportion of users in the peri-urban area of Sultanabad, compared to Garden (Table 1). Moreover in this area, the people were relatively less educated compared to the people in Garden area (Table 2).

In these 2 samples, 16% had a history for coronary artery disease (CAD), while diabetes mellitus was reported to be 7%. A total of 28 (4%) subjects had reported to be suffering from depression, from which 21 (75%) were using benzodiazepines. Among the recruited subjects, 36 reported to have seen a psychiatrist before the interview. From those, 22 were using benzodiazepines.

### Data on users

The most used benzodiazepine was bromazepam (29%), followed by diazepam (26%), alprazolam (24%) and lorazepam (24%). The median duration of primary use was 144 weeks (IQR = 48-240)). About 67% reported to have been using benzodiazepines continuously; 46% of them reported to have been using these daily, and 21% were using it on a weekly basis. We estimated the median diazepam equivalent dose to be 10 mg (IQR = 5-25). A small proportion had been using on the advice of a psychiatrist (3%), while majority had been using these upon either the prescription of local practitioners (40%) or self (24%). Only 22% of the users were taking these for genuine complaints, especially depression, while most were using for minor complaints such as insomnia (21%) and anxiety (8%).

Using applied logistic regression, crude estimates for odds ratios for educational categories revealed that the odds of benzodiazepine use were more among less educated subjects (Table 1). In the category of occupation, crude estimates revealed the likelihood of greater use among the unemployed, retired, housewives and salaried worker categories (Table 1). In the adjusted model, increasing age, location of residence, female sex, unemployment and psychiatric consult were associated with increase in odds of benzodiazepine use in the community (Table 3).

**Table 3:** Adjusted odds ratios (with 95% CI) computed using multiple logistic regression

Characteristics		Wald χ^2^	df	P-value	Adjusted OR (95% CI)
**Age**		29.9	1	0.001	1.06 (1.04-1.09)

**Location**	Garden				1
	Sultanabad	25.3	1	0.001	4.49 (2.50-8.10)

**Gender**	Male				1
	Female	4.0	1	0.04	2.20 (1.02-4.75)

**Marital status**	Unmarried				1
	Married	3.0	1	0.08	2.39 (0.89-6.40)

**Occupation**	Student				1
	Professional	0.03	1	0.86	0.89 (0.20-4.08)
	Housewife	0.36	1	0.55	0.61 (0.12-3.18)
	Retired	0.25	1	0.62	1.66 (0.24-11.67)
	Labor	0.13	1	0.71	0.68 (0.14-3.36)
	Business	0.93	1	0.33	0.39 (0.06-2.78)
	Unemployed	4.9	1	0.03	14.50 (1.38-151.87)

**Psychiatric consult**	No				1
	Yes	40.8	1	0.001	18.9 (7.68-46.65)

df = degrees of freedom; OR = Odds ratio; CI = Confidence interval

## Discussion

The purpose of this study was to estimate the frequency of benzodiazepine use among the 2 local communities in Karachi, Pakistan. To the best of our knowledge, no study of this kind has been conducted in this region.

The proportionate benzodiazepine use among the studied sample was 14%; higher than that reported in Boston, France and Lebanon [26-28]. One reason for this high estimate could be the unregulated availability of benzodiazepines in the local market. Additionally, in presence of co-morbidities such as hypertension, general practitioners do prescribe benzodiazepines for better control and relief. Our study showed that about 40% of cases with co-morbidities had been using benzodiazepines on the advice of general practitioners. The reported high estimates point to the need to caution both doctors and patients on the associated risk of dependence, and to regulate the accessibility of benzodiazepines in this country.

Benzodiazepine users in our sample were mostly elderly females. This finding is quite consistent with the findings by other researchers [22,24,27-29,32-36]. Older people are more likely to be using benzodiazepines for relieving insomnia and anxiety related to isolation, depression and medical ailments [37,38]. Reports also suggest that anxiety occurs more in females compared to males [36,39]. Moreover, as females tend to live longer than males, the psychological problems are more likely to be encountered by them [40]. Another important fact is that females, in general, are more cooperative in explaining their health problems and thus are relatively more compliant towards the prescribed medications [40,41].

Bromazepam was found to be the most used benzodiazepine in the study settings. Similar results have also been reported in community based studies in Brazil [24,42]. The second most commonly used benzodiazepine was diazepam. The median duration of primary use in our study was 144 weeks, whereas the recommended duration by clinicians/psychiatrists should not be more than 4 weeks [43]. According to literature, caution should be exercised while prescribing benzodiazepines; as in case of those with shorter half lives and high potency, there is a tendency for therapeutic dose dependence; while those with longer half lives are usually associated with impaired cognition and prolonged sedation, as the drug may get accumulated in the body of the individual [44-47]. In Pakistan, the situation is quite serious due to the easier access of these drugs over-the-counter. This may pose a risk to the general population.

In this research, we found the use of benzodiazepines more among the less educated class of subjects. Other researchers have also shown the maximum use of benzodiazepines among the less educated individuals [32,36,48]. Awareness regarding the risks associated with benzodiazepine use among the educated individuals could be a plausible reason for restricted use of these drugs. In our study, this was not significant in the multivariable model, perhaps due to stratification of the educational categories. The adjusted regression model showed that the unemployed persons were more inclined to use benzodiazepines. Similar findings have been also reported by other investigators, such as Lagnaoui et al. who have indicated higher rates of benzodiazepine use among jobless individuals [27].

Our results should be seen within the context of certain limitations of this research. This study being the first of its kind in this country, describes the "current" status of benzodiazepine use. Another limitation could be fewer subjects in the occupational category for unemployment (1.3%); which, though significant, we believe could be "under-reporting." In low income communities a reasonable proportion of population might have been working for just 1-2 days in a week and considering themselves as employed. We presented the results for 2 different communities in Karachi and these 2 sites would not be representative of the whole city of 18 million people. Nonetheless, we believe the results to be fairly representative of the concerned populations owing to the systematic random sampling employed in the research and a reasonable sample size. The findings we present are consistent with what has been reported by other researchers on Western populations. More surveys at multiple communities would provide a deeper insight into the prolonged use of benzodiazepines and various factors governing its usage, in Pakistan. This would provide the necessary sensitization of the medical community and measures to be taken to effectively control benzodiazepine use in the developing world.

## Conclusion

The proportion of benzodiazepine use in the 2 communities of Karachi, Pakistan is relatively higher than that reported in the developed countries. It is a matter of concern in view of the associated adverse events and the prevalent physical and psychological conditions in developing countries. Further research at the national level would be imperative to provide a deeper insight into the problem of prolonged benzodiazepine use especially without a proper consult of a psychiatrist (only 4.7% in the study sample had been seen by a psychiatrist and among the benzodiazepine users nearly 40% had been using it without a psychiatric consult) and its associated mental health issues. Sensitization of the medical community and the masses to this growing problem of benzodiazepine use and associated risks is necessary to control it in the developing societies.

## Competing interests

The authors declare that they have no competing interests.

## Authors' contributions

SA, SPI and RAMK were involved in the conceptualization and implementation of the research. They were also involved in data interpretation and write up of the manuscript. SPI provided epidemiological support and performed the statistical analyses. SF, YP and AT were involved in implementation of the research and data interpretation. MZ was involved in data entry, cleaning and interpretation. All authors have read and approved the manuscript.
